# The relationship between patients' experiences of continuity of cancer care and health outcomes: a mixed methods study

**DOI:** 10.1038/sj.bjc.6604164

**Published:** 2008-01-29

**Authors:** M King, L Jones, A Richardson, S Murad, A Irving, H Aslett, A Ramsay, H Coelho, P Andreou, A Tookman, C Mason, I Nazareth

**Affiliations:** 1 Department of Mental Health Sciences, Royal Free & University College Medical School, Hampstead Campus, Rowland Hill Street, London NW3 2PF, UK; 2 Marie Curie Palliative Care Research Unit, Department of Mental Health Sciences, Royal Free & University College Medical School, Hampstead Campus, Rowland Hill Street, London NW3 2PF, UK; 3 King's College London, Florence Nightingale School of Nursing and Midwifery, Waterloo Bridge Wing Franklin Wilkins Building, 150 Stamford Street, London SE1 9NH, UK; 4 St Joseph's Hospice, Mare Street, Hackney, London E8 4SA, UK; 5 MRC General Practice Research Framework and Department of Primary Care & Population Science, Royal Free and University College Medical School, Hampstead Campus, Rowland Hill Street, London NW3 2PF, UK

**Keywords:** continuity of care, cohort study, cancer, service delivery, transitions in care

## Abstract

It is difficult to define continuity of care or study its impact on health outcomes. This study took place in three stages. In stage I we conducted qualitative research with patients, their close relatives and friends, and their key health professionals from which we derived a number of self completion statements about experienced continuity that were tested for reliability and internal consistency. A valid and reliable 18-item measure of experienced continuity was developed in stage II. In stage III we interviewed 199 patients with cancer up to five times over 12 months to ascertain whether their experiences of continuity were associated with their health needs, psychological status, quality of life, and satisfaction with care. The qualitative data revealed that experienced continuity involved receiving consistent time and attention, knowing what to expect in the future, coping between service contacts, managing family consequences, and believing nothing has been overlooked. Transitions between phases of treatment were not associated with changes in experienced continuity. However, higher experienced continuity predicted lower needs for care, after adjustment for other potential explanatory factors (standardised regression coefficients ranging from −0.12 (95% CI −0.20, −0.05) to −0.32 (95% CI −0.41, −0.23)). Higher experienced continuity may be linked to lower health care needs in the future.

Since 2000 (Department of Health, 2000) cancer care has been the subject of UK government targets to reduce waiting times for diagnosis and treatment (Department of Health, website:www.performance.doh.gov.uk/cancerwaits), the European Working Time Directive has changed patterns of working and general practitioners no longer provide out of hours cover for patients. These changes impact on continuity of care, which is a key research priority for the Cancer Reform Strategy (Department of Health, 2006, 2007). The National Institute for Health and Clinical Excellence guidance on supportive and palliative care recommends promotion of continuity of care and taking account of patients' and carers' views (NICE guidance on palliative and supportive care for adults with cancer, 2004).

## Conceptualising continuity of care

Continuity of care is not easy to define or measure. It involves consistent information, cross boundary and team continuity, flexibility in response to need, care from as few professionals as possible, and a main contact person (Freeman *et al*, 2000). More recently, informational, management, and relational continuity have been described (Centre for Health Services and Policy Research, 2006) (Box 1). Haggerty *et al* (2003) defined interpersonal continuity in terms of the doctor–patient relationship but regarded it as difficult to measure and were uncertain that it improves care. Furthermore, we know little about how patients experience continuity of care and whether this affects their health outcomes (Haggerty *et al*, 2003). Cancer will affect one in three people at some time in their lives. For most cancers, 5-year survival has been improving steadily over the last 30 years, especially breast and bowel cancer (Coleman, 1999). As a result, cancer is becoming a chronic illness with which patients must both live and face the threat of recurrence. Cancer treatments are often intensive and disruptive to patients' lives. Typical patients encounter several different layers of care during their illness, especially if treatment continues over a protracted period. Thus continuity is a crucial issue in this field of medical care

Our main questions were: can continuity of cancer care be defined from the service user's perspective and do their perceptions of continuity affect their health outcomes? We aimed to (1) use qualitative research to understand how patients and people close to them experience continuity of care; (2) apply key concepts arising from the qualitative data to develop a measure of continuity; and (3) evaluate if patients' experiences of continuity of care determine their health outcomes.

## MATERIALS AND METHODS

This mixed methods study took place in three stages. In stage I, we conducted qualitative research with patients, close persons, and health professionals. In stage II, these data were used to derive a standardised measure of experienced continuity. In stage III, we used this measure in a cohort of patients with cancer to ascertain whether continuity of care was associated with their health needs, psychological status, quality of life, and satisfaction with care. Patients were diagnosed with breast, lung or colorectal cancer. These tumour sites were chosen as they are commonly occurring and provided an overall gender balance. Sampling was purposive and we aimed to recruit equal numbers from each cancer type at each of five treatment phases (Morse and Fife, 1998; Farell and Lewis, 2000):

1. Initial diagnosis defined as within 4 weeks of receipt of diagnosis and before specific treatment begins
2. End of first treatment when first treatment is defined as the treatment plan agreed by the treating clinical team
3. Remission defined as having been clinically disease-free for a minimum of 6 months
4. Relapse defined as first recurrence of cancer of original cell type leading to re-presentation to secondary services
5. Referral to specialist palliative care, which may occur at any time during illness, but usually when symptoms are severe or the terminal phase is anticipated.

### Stage I: qualitative study

We obtained ethical approval from a Local Research Ethics Committee and the study was conducted over eight months in 2002 and 2003. We recruited participants from five general practices across north London to reflect as wide a range of socio-demographic variability as possible. Recruiting from primary care, we aimed to reach patients who might have been lost to follow-up in secondary care. Practise staff used electronic records to identify patients with lung, colorectal, and breast cancers at each treatment phase and obtained consent to pass their details to the research team. Patients and close persons were interviewed by AI and HA in their homes or the general practise according to their wishes. At first research contact, patients nominated one close person and up to two key health professionals who might also take part. We intended to place most emphasis on the views of patients and close persons but also interviewed key professionals where possible to understand how these differed (Nazareth *et al*, in press). We avoided the term ‘continuity of care’ in interviews, attempting instead to focus on consistent services and to encourage patients to reflect on issues that captured the balances needed to ensure that patients could access diagnosis, treatment, and follow-up while maintaining a sense of normality and hope for the future. Interviewers used a set of prompts that were modified in response to piloting. Health-care professionals were also asked how continuity of cancer services might be improved. We aimed to recruit until no new themes emerged from the data or we reached a maximum of 30 patients.

### Analysis of qualitative data

Interviews were read several times independently and coded by two researchers; there was a mean rate of disagreement in codes of 9.1%, which were settled by consensus. The codes were indexed and transferred to QSR NVivo v1.2 for framework analysis (Neuendorf, 2002). Data were discussed repeatedly at research team meetings and a collective decision was taken to stop interviewing after 28 patients had been recruited as no new themes were emerging. The detailed results of this qualitative study are reported elsewhere (Nazareth *et al*, in press). Here we report how the qualitative data were used to construct a number of statements on experienced continuity of care that were standardised and used in our cohort study.

### Stage II: development of the measure of experienced continuity

MK, LJ, and IN who have research and clinical experience in cancer and AI and HA who conducted many of the qualitative interviews discussed each theme in the qualitative data. We used the Haggerty model (Haggerty *et al*, 2003) as a theoretical framework in seeking to establish a set of statements from the qualitative analysis that related to themes of continuity. We kept the instrument brief (20 statements) and circulated it to the project steering committee and clinicians for further modification. On the advice of user representatives we used a Likert format with five responses to each statement ranging from ‘strongly agree’ to ‘strongly disagree’. We evaluated test-retest reliability of the 20-item instrument by asking 38 patients with a range of cancers in different stages in treatment (who were not part of the cohort described below) to complete it on two occasions 2–3 weeks apart. Internal consistency of the statements was estimated in the baseline cohort data.

### Stage III: cohort study

We undertook the cohort study in three National Cancer Networks in London between May 2003 and August 2005. Sampling was purposive to ensure consistency of recruitment across the three networks. Staff approached patients with breast, lung, or colorectal cancer in the five phases of treatment and sought consent to pass their details to the research team. Recruitment was assisted by National Cancer Research Network research staff. After giving consent for participation in the cohort, patients undertook assessments in their own homes. Each patient provided information on their socio-demographic characteristics, type of cancer, treatment phase, and cancer service network. They then completed the 20 statements developed on experienced continuity and a number of other measures:

1. *Needs for care*: the Supportive Care Needs Survey (Bonevski *et al*, 2000; Steginga *et al*, 2001) measures physical and daily living, psychological, health system and information, sexuality, and patient care and support needs for care.
2. *Quality of life* was measured using the Euroqol (EuroQol Group, 1990).
3. *Psychological status* was assessed using the 28-item General Health Questionnaire (Goldberg and Williams, 1988).
4. *Satisfaction*: As there was no standardised measure of satisfaction with cancer services, we developed visual analogue scales for satisfaction with overall care, continuity of care, supportive care, information needs, and quality of communication (scored 0 to 10 towards higher satisfaction). The five scales were summed to a total score.

All participants completed these measures again at face-to-face assessments, 3, 6, 9, and 12 months later. The interview was piloted with a number of patients prior to embarking on the continuity study and modifications made. Research personnel changed slightly throughout the study and so occasionally patients would be interviewed by a different researcher at a follow-up point. All the interviews were carried out by AI, HA, AR, HC, and PA. Ethical approval was obtained from a Multicentre Ethics Committee.

### Statistical analysis of the quantitative research and sample size

We conducted all analyses using Stata release 9.

#### Reliability of the continuity statements

We estimated test-retest reliability in our separate reliability study by condensing scores for each statement (1 for each affirmation of continuity and 0 for other responses) and calculating the intra-class correlation coefficient. Internal consistency was estimated in patients in the cohort study using Cronbach's alpha.

#### Analysis of the cohort

We explored the heterogeneity of the cohort data by testing whether patient experiences were equivalent in each phase of treatment, regardless of the time point, using the *χ*^2^ test for categorical and F test for continuous variables. We undertook a multilevel model analysis with two levels (time period nested within patients) and a random intercept. We estimated the parameters in the multilevel model using the maximum likelihood method. Cancer networks and centres were included as covariates in the regression models. Treatment phase, cancer network, treatment site, follow-up point, and transition from one treatment phase to another were significantly associated with missing data on our main outcomes and were adjusted for in our regression models. Multiple imputation of missing data by chained equation method was used to derive five imputed data sets (Royston, 2004, 2005a, 2005b; van Buuren *et al*, 2006). We fitted a multilevel model to each of the five sets, averaging regression coefficients and using Rubin's rule to obtain the correct s.e. (Schafer, 1999). We did not impute missing demographic data as these could be carried forward from baseline. We used a time lag predictive model in which experienced continuity score is related to each outcome measure one time point later.

#### Sample size for the cohort study

We could not estimate *a priori* the size of any association between our measure of experienced continuity and our main outcomes; thus we aimed to recruit 250 patients, given the funding constraints of the study. We can however, estimate the statistical soundness of our analysis. Since several regression models were fitted, the size of the data varied due to missing values. There were at least 337 observations available for these analyses. Using rule of thumb of 10 participants for each variable (Harrell, 2001) and inflating the sample size required due to the presence of repeated measurements by 1.32, 160 participants are required to estimate up to 12 coefficients in a model. The inflation factor was estimated using an average cluster size of approximately two (337 observations/199 participants) and the largest intra-cluster correlation coefficient was (ICC) estimated from the data of 0.32 (mean ICC 0.04).

## RESULTS

### Stage I: participants in the qualitative study

Twelve were women with breast cancer (mean age 58 years), nine had colorectal cancer (five women, mean age 67 years), and seven had lung cancer (four women, mean age 71 years). Twenty-four nominated a close person, of whom 18 agreed to interview; eight were nominated by patients with breast cancer, five by those with colorectal cancer, and five by those with lung cancer. Seven were spouses or partners, five were adult daughters, five were friends, and one was a colleague. Sixteen patients nominated at least one key professional; 13 nominated general practitioners, and 10 nominated secondary care professionals, seven of whom were nurses (see Nazareth *et al*, in press for further detail).

### Themes in the qualitative analysis

Patients talked mostly about consistency of services, whether service providers had a collective memory about their care, and how they coped between service contacts. Unlike the professionals who focused mainly on the structure of services, patients focused on how well they were known to services and what to expect in the future. All patients, close persons and professionals emphasised receiving sufficient time and attention from services and ensuring nothing had been overlooked. Patients and close persons wanted to be well informed about treatments and side effects. Managing the impact of treatments and maintaining a feeling of normality were crucial. Several patients indicated their need at times for discontinuity by wanting to forget the illness and achieve a sense of normality. Family roles affected patients' and close persons' attitudes to illness and in turn their sustained engagement with care. How services responded and what professionals remembered about them influenced their perceptions of continuity. Patients were generally accepting of their care, whereas close persons expressed more concern at perceived delays and lacked confidence in service providers. General Practitioners found themselves advising on future treatment decisions and the likely course of illness, as well as chasing up appointments with secondary care. Clinical nurse specialists were aware of their key role for patients, particularly with regard to trust and continuity (Nazareth *et al*, in press).

### Stage II: the measure of experienced continuity

We condensed the qualitative themes into a 20-item instrument. However, two statements were eventually discarded as they could not apply to all patients (one on home social services and one on social security benefits). The 18 items remaining (Box 2) had high internal consistency at baseline in the prospective cohort data (Cronbach's alpha 0.87) and no single item removal improved on this. Scores on questions 1, 3, 5–7, 9, 10, 12, 13, and 17 were reversed so that a high total score indicated high experienced continuity.

Thirty-eight patients entered into the separate test-retest reliability study of whom 30 (20 women) (79%) completed it a second time. Of the latter, eight patients had breast cancer, eight colorectal, four lung and one brain cancer, and nine were receiving palliative care for a range of cancers. Their mean age was 62.2 years (s.d. 10.6). The intra-class correlation for total continuity score was 0.82 (95% confidence intervals (CI) 0.70, 0.94) indicating good reliability.

### Stage III: prospective cohort study

#### Response rates and characteristics of the population at recruitment

**Figure 1:**
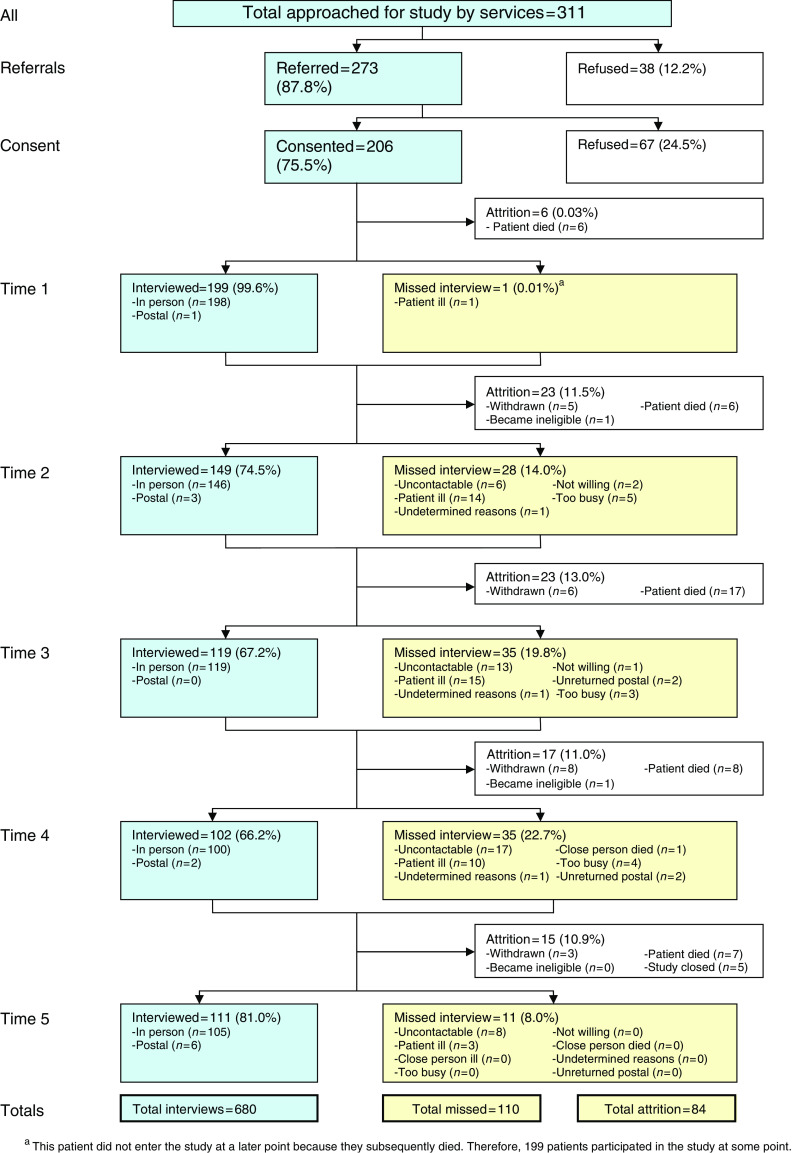
Flow diagram study.

Two hundred and seventy-three patients (103, 78, and 92 in each cancer network), allowed their details to be passed to the research team. When contacted 199 (73%) agreed to participate and 74 refused. We could not record data on those who refused for ethical reasons. Participants lost at one follow-up point occasionally returned to the cohort at a subsequent point (Figure 1). As expected, there was considerable attrition due to death.

#### Clinical, psychological, and spiritual status at recruitment

There were no socio-demographic differences between patients in the five treatment phases at baseline (Table 1). Eighty-three per cent of participants reported that they had a main contact person in the cancer services. Other principal differences were: (1) lowest needs for care during remission; (2) lowest quality of life after relapse and during palliative care; and (3) best psychological status in remission and worst at relapse (Table 2). Satisfaction with services was somewhat higher in remission. Mean scores on experienced continuity did not vary with treatment phase or type of cancer. This lack of heterogeneity in our main variable confirmed that the data could be analysed as a whole.

#### Experienced continuity and health outcomes over 12 months

Eighty-one (41%) people made at least one transition between treatment phases, mostly between diagnosis, first treatment, and remission. One person moved back from palliative care into remission and four moved from first relapse into a further remission. Experienced continuity of care did not change significantly in the whole cohort over time (per follow-up point, standardised regression coefficient B −0.01, CI −0.06, 0.03). There was also no significant association between continuity and a transition from one treatment phase to another (standardised B −0.25, CI −0.51, 0.01). After adjustment for potential confounding influences, mean continuity score at one time point was negatively predictive in the complete case and imputed data sets of all subscales of the Supportive Care Needs Survey at a forthcoming time point (Table 3). This means that higher experienced continuity at one time point predicted lower needs for supportive care at the next. The result for the association between continuity and satisfaction was not replicated in the imputed data (Table 3) and therefore needs to be interpreted with caution.

A complex non-linear relationship was observed between the health outcomes measured by the Euroqol and General Health Questionnaire, and the needs identified in the Supportive Care Needs Survey. This may go some way to explain why continuity of care was not directly associated with these health outcomes.

## DISCUSSION

### Main findings

Rather than adopting a top-down theoretical approach to continuity, we used a bottom-up empirical approach to explore how continuity is experienced by patients. Experienced continuity is a concept involving consistent time and attention, knowing what to expect in the future, coping between service contacts, managing family consequences and believing nothing in treatment has been overlooked. Our measure of experienced continuity had validity based on patients', close persons' and health professionals' grass-roots opinions, and the total score has acceptable test-retest reliability. Eleven statements concern some of the issues that professionals face in delivering continuity of care (statements 1–8 and 16–18). Transitions between phases of treatment have no impact on continuity but high experienced continuity of care is predictive of fewer future needs for supportive care.

### Strengths and limitations of the study

Some conceptual overlap between continuity and quality of care is possible as services that offer good continuity are often of higher quality. Although there are similarities in content between some items in our measure of experienced continuity and those in the Supportive Health Needs Survey's subscale health system and information needs, this is not the case for the other subscales. Residual confounding in our time-lag analysis is possible and, given the repeated measures of our analysis, it is likely that we are overfitting the estimates of the association between experienced continuity and health outcomes. Nevertheless, our multilevel approach helped to reduce this latter possibility. Our cohort recruitment fell short of 250, mainly because people who indicated they would participate subsequently changed their minds. Attrition due to death or incapacitating illness was the main limitation of the study. Imputation methods are currently limited within multilevel, repeated measures analyses because they assume independence of the observations. Thus we regard our imputed data as a sensitivity analysis to be as cautious as possible. Although there was some consistency in the research staff conducting interviews, during the course of the three stages of the work, all research assistant personnel changed. We cannot therefore comment on the possible effects on experienced continuity of the conduct of the research itself. Finally, although covering areas with varying socio-economic conditions and services, it may not be possible to generalise our findings beyond London.

### Experienced continuity

Experienced continuity of care will not simply mirror continuity as delivered by health professionals. It is a measure of the outcome rather than the delivery of care. In our study, patients experienced consistent care in terms of sufficient knowledge about the cancer and its prognosis, a perception of ready access to services, confidence that they can manage when not in touch with services and assurance that their families can cope. Coping between appointments may mean patients can visualise in their mind's eye a stable and consistent service. Good family relationships with few secrets may mean that communication with professionals is facilitated. Patients in our qualitative study seldom mentioned communication between services or a key person to contact. However, patients and close persons are often not aware of communication between services and may take a key professional for granted. As the patients in the cohort confirmed, key contact professionals are already a fundamental part of London's cancer services.

### Providing continuity

Rather than something professionals offer, continuity is an interaction between the care setting, the professionals' management of it, and patients' beliefs about and attitudes to those close to them. For example, if patients have difficulty talking about cancer to their close friends and family and do not involve them in their treatments, continuity of care may be placed in jeopardy. How patients cope between appointments is linked to the concept of self-management and how to engage patients in their healthcare (Coulter, 2006).

### Does experienced continuity matter?

Most evidence on the impact of continuity concerns the delivery of single interventions such as the availability of a consistent professional. Considering other diseases using this concept of delivered continuity, there is evidence for (Parchman *et al*, 2002) and against (Gulliford *et al*, 2006) a relationship with better glycaemic control (Parchman *et al*, 2002) in diabetes care. Enhanced continuity may also improve outcomes in obstetric care, albeit at the risk of unnecessary interventions (Hodnett, 2000), while a consistent clinician and site for treatment are associated with higher rates of uptake of screening for cervical cancer (O'Malley *et al*, 1997). Continuous primary care provision also leads to earlier detection of cancer (Reid and Rozier, 2006). Our study in cancer is the first to suggest that the degree to which patients experience continuity may reduce their needs for care. This is particularly the case for health information needs (Table 3), which, given the content of our continuity instrument, is understandable. Continuity was not linearly associated with later quality of life or psychological status. Although it is possible for the quality of experienced continuity to have an impact on future health status, our observation of the complex relationship between health needs and health outcomes, and the likely role of other factors such as disease progression, indicate that this relationship is complex and requires further research.

### Transitions in treatment

It is reassuring that transitions between phases of treatment were not associated with changes in experienced continuity. Although clinical experience suggests that transitions, such as cessation of active treatment and referral to specialist palliative care, might cause a sense of abandonment, we found no such impact on experienced continuity. It is unlikely that our measure of experienced continuity lacked sensitivity to such change, given its demonstrated links with perceived needs for care over time.

### Conclusions

Our findings suggest that patients' experience of continuity is an outcome of service delivery that can be facilitated rather than provided. We need to avoid narrow, service-based concepts of continuity if we are to understand its impact on health outcomes. Higher experienced continuity may be associated with lower health care needs in the future. Our findings require confirmation as well as reflection on how we might ensure that patients' experiences of continuity are as positive as possible. We have already begun the first phase of research into the development, piloting, and testing in a feasibility randomised controlled trial of an intervention to enhance continuity of care in cancer. The intervention focuses on enabling patients to change and improve their experiences of continuity. Such work also requires application in other areas of chronic care.

## Figures and Tables

**Table 1:** Patients' characteristics by phase of treatment at recruitment

				**Treatment phase**															
	**All (*N*=199)**			**Initial diagnosis (*N*=45)**			**Completion first treatment (*N*=48)**			**Remission (*N*=46)**			**Relapse (*N*=27)**			**Specialist palliative care (*N*=33)**			
	***N***	**Mean**	**s.d.**	***N***	**Mean**	**s.d.**	***N***	**Mean**	**s.d.**	***N***	**Mean**	**s.d.**	***N***	**Mean**	**s.d.**	***N***	**Mean**	**s.d.**	***P*-value**
Age in years	191	61.2	11.8	45	60.0	11.1	43	60.3	11.1	45	61.2	11.6	25	61.7	13.2	33	63.2	13.4	0.810
	*N*		%	*N*		%	*N*		%	*N*		%	*N*		%	*N*		%	
*Gender*																			
Male	63		31.7	14		30.4	15		32.6	13		27.7	11		42.3	10		29.4	0.765
Female	136		68.3	32		69.6	31		67.4	34		72.3	15		57.7	24		70.6	
																			
*Marital status*																			
Single	35		17.6	11		23.9	7		15.2	8		17.0	6		23.1	3		8.8	
Married/cohabiting	121		60.8	27		58.7	30		65.2	24		51.1	17		65.4	23		67.7	0.190
Formerly married^a^	41		20.6	8		17.4	7		15.2	15		31.9	3		11.5	8		23.5	
																			
*Household (lives with)*																			
Alone	56		28.1	15		32.6	11		23.9	18		38.3	5		19.2	7		20.6	
Spouse/partner only	77		38.7	15		32.6	15		32.6	16		34.0	13		50.0	18		52.9	0.520
Anyone else	62		31.2	15		32.6	19		41.3	12		25.5	7		26.9	9		26.5	
																			
*Socio-economic class*																			
Group A^b^	111		55.8	18		39.1	29		63.0	27		57.5	15		57.7	22		64.7	0.137
Group B^c^	74		37.2	22		47.8	16		34.8	18		38.3	10		38.5	8		23.5	
																			
*Ethnicity*																			
White British	150		75.4	33		71.7	36		78.3	36		76.6	19		73.1	26		76.5	
Any other White background	18		9.1	4		8.7	3		6.5	6		12.8	2		7.7	3		8.8	
Black/Black mixed background	15		7.5	5		10.9	2		4.4	2		4.3	3		11.5	3		8.8	0.497
Asian/Asian mixed background	6		3.0	1		2.2	0		0.0	3		6.4	2		7.7	0		0.0	
Any other background	8		4.0	3		6.6	4		8.7	0		0.0	0		0.0	1		2.9	

a Formerly married=separated, divorced or widowed.

b Group A=Socio-economic classes I, II and III non-manual.

c Group B=Socio-economic classes III manual, IV, V and housewife/househusband.

**Table 2:** Scores on experienced continuity and health and service outcomes stratified by phase of cancer treatment at recruitment

				**Treatment phase**															
	**All (*N*=199)**			**Phase 1 diagnosis (*N*=46)**			**Phase 2 completion first treatment (*N*=46)**			**Phase 3 In remission (*N*=47)**			**Phase 4 On relapse (*N*=26)**			**Phase 5 Specialist palliative care (*N*=34)**			
	***N***	**Mean**	**s.d.**	***N***	**Mean**	**s.d.**	***N***	**Mean**	**s.d.**	***N***	**Mean**	**s.d.**	***N***	**Mean**	**s.d.**	***N***	**Mean**	**s.d.**	***P*-value**
*Continuity of care*																			
Total score on 18 statements (range: 0–72)	181	51.8	9.9	45	52.1	9.3	41	50.8	12.9	41	52.1	7.9	24	53.2	6.1	30	51.0	11.4	0.879
																			
*Satisfaction*																			
Satisfaction (range: 0–50)	191	41.7	8.8	42	41.5	8.8	46	39.3	9.9	46	44.0	6.3	26	43.6	6.8	31	40.8	10.8	0.086
																			
*SCNS*																			
SCNS Physical and daily living needs (range 0–100)	194	11.7	4.7	44	12.0	4.4	45	11.6	4.4	46	9.4	4.1	27	12.7	5.2	33	13.4	4.6	0.001
SCNS Psychological needs (range 0–100)	186	21.4	8.8	44	21.3	9.5	44	22.8	7.9	45	18.6	7.9	25	25.0	10.7	28	20.6	7.2	0.037
SCNS Patient care+support needs (range 0–100)	193	11.6	3.7	44	12.8	4.3	46	12.2	4.2	47	9.9	2.7	26	11.7	3.3	30	11.6	2.9	0.003
SCNS Health system +information needs (range 0–100)	184	21.8	6.9	42	23.8	8.2	46	24.5	8.4	47	19.1	4.9	25	21.9	3.7	24	19.5	4.6	<0.001
SCNS Sexuality needs (range 0–100)	193	4.3	2.4	44	4.7	2.3	45	4.0	2.0	47	4.3	3.1	25	4.3	3.1	32	4.2	2.2	0.727
																			
*Euroqol*																			
Euroqol ED5D (range: −0.59–1.00)	196	0.67	0.5	46	0.73	0.23	46	0.73	0.23	47	0.78	0.13	26	0.64	0.28	31	0.36	1.1	0.001
Euroqol thermometer (range 0–100)	190	66.6	18.9	43	64.3	20.7	45	69.5	16.4	45	74.8	15.8	26	60.8	20.3	31	58.7	18.1	0.001
																			
*GHQ*																			
GHQ Total (range 0–28)	173	6.3	0.4	43	7.2	0.8	39	6.9	0.9	43	4.1	0.8	20	8.5	1.5	28	5.7	0.9	0.01
GHQ caseness^a^																			
Non case	92 (53.2%)			16 (37.2%)			22 (56.4%)			31 (72.1%)			9 (45.0%)			14 (50%)			0.022
Case	81 (46.8)			27 (62.8%)			17 (43.6%)			12 (27.9%)			11 (55.0%)			14 (50%)			
																			

a Caseness defined at a threshold of 5/6.

**Table 3:** Impact of experienced continuity score on health and service outcomes^a^

	**Complete data**	**Imputed data^b^**
	**Standardised regression coefficient (CI)**	**Standardised regression coefficient (CI)**
*Supportive care needs survey subscale scores*		
Physical and daily living needs	−0.16 (−0.24, −0.08)	−0.19 (−0.36, −0.01)
Psychological needs	−0.14 (−0.24, −0.05)	−0.15 (−0.27, −0.03)
Health system and information needs	−0.32 (−0.41, −0.23)	−0.28 (−0.39, −0.17)
Sexuality needs	−0.12 (−0.20,−0.05)	−0.19 (−0.31, −0.07)
Patient care and support needs	−0.20 (−0.29, −0.11)	−0.15 (−0.25, −0.05)
		
*Euroqol*		
Euroscore	−0.05 (−0.14, 0.03)	0.04 (−0.08, 0.16)
Thermostat	0.09 (−0.01, 0.19)	0.07 (−0.04, 0.18)
		
*General health questionnaire 28*		
Total score	−0.04 (−0.13, 0.05)	−0.06 (−0.16, 0.04)
		
*Satisfaction with services*		
Total score	0.04 (−0.06, 0.14)	−0.15 (−0.24, −0.06)

a After adjustment for baseline outcome, treatment phase, treatment site, network, period (time of interview) and transition from one treatment phase to another.

b Mean multilevel model regression coefficients arising from the five imputed data sets.

CI=95% confidence intervals.

Figures in blue indicate significant effects.

## **Box 1** Types of continuity

*Informational continuity*—The use of information on past events and personal circumstances to make current care appropriate for each individual

*Management continuity—*A consistent and coherent approach to the management of a health condition that is responsive to a patient's changing needs

*Relational continuity*—An ongoing therapeutic relationship between a patient and one or more providers

## **Box 2** Statements on experienced continuity of care

**Item no.** **Items**

1 I have received enough time and attention from the cancer services

2 I do not see the cancer services often enough

3 I am getting consistent information about my illness from health care staff

4 I frequently have to chase up cancer services to get things done

5 I have been well informed about what my treatment will involve over the next few months

6 I am aware of what side-effects to expect from my cancer treatments

7 I have been told what to expect in terms of my overall health over the next few months

8 I feel out of touch with the cancer services between appointments

9 I feel able to cope with minor complications that may arise

10 I am coping well between my appointments with the cancer services

11 I have difficulty accepting the limitations my health places on my life

12 I feel supported by the people closest to me

13 I feel my friends and relatives are able to help me cope with my illness

14 I am worried about the emotional state of the people closest to me

15 I feel I depend too much on my friends or relatives

16 I have received some misleading information from the cancer services

17 I am content that I have received a full medical examination with regard to my cancer

18 I am worried that some things may have been overlooked
